# Arbitrary lattice symmetries via block copolymer nanomeshes

**DOI:** 10.1038/ncomms8448

**Published:** 2015-06-23

**Authors:** Pawel W. Majewski, Atikur Rahman, Charles T. Black, Kevin G. Yager

**Affiliations:** 1 Center for Functional Nanomaterials, Brookhaven National Laboratory, Upton, New York 11973, USA

## Abstract

Self-assembly of block copolymers is a powerful motif for spontaneously forming well-defined nanostructures over macroscopic areas. Yet, the inherent energy minimization criteria of self-assembly give rise to a limited library of structures; diblock copolymers naturally form spheres on a cubic lattice, hexagonally packed cylinders and alternating lamellae. Here, we demonstrate multicomponent nanomeshes with any desired lattice symmetry. We exploit photothermal annealing to rapidly order and align block copolymer phases over macroscopic areas, combined with conversion of the self-assembled organic phase into inorganic replicas. Repeated photothermal processing independently aligns successive layers, providing full control of the size, symmetry and composition of the nanoscale unit cell. We construct a variety of symmetries, most of which are not natively formed by block copolymers, including squares, rhombuses, rectangles and triangles. In fact, we demonstrate all possible two-dimensional Bravais lattices. Finally, we elucidate the influence of nanostructure on the electrical and optical properties of nanomeshes.

## Abstract (web summary)

Nanoscale patterning methods based on self-assembly promise to revolutionize the fabrication of high-tech devices, but suffer from a limited number of possible lattice symmetries. Here, the authors use a laser zone annealing technique to pattern block copolymers into any 2D mesh motif they desire.

## Graphical abstract

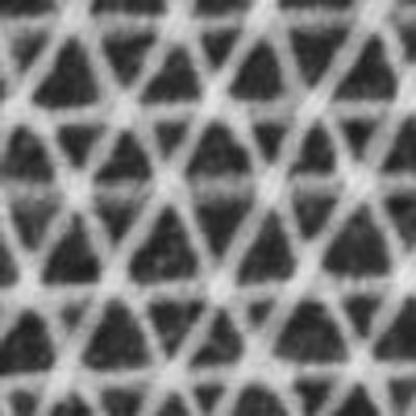

Block copolymers (BCPs) are a powerful self-assembly motif, wherein the chemically distinct blocks of a polymer architecture microphase separate into a well-defined nanoscale morphology. BCP self-assembly enables the formation of nanoscale structures over macroscopic areas, with the unit cell defined by polymer architecture^1^,^2^. These spontaneously formed nanostructures show great promise as functional materials^3^,^4^. The symmetry and unit cell of a self-assembled lattice is dictated by energy minimization criteria. This is, of course, the guiding principle of self-assembly. And yet, this inherently gives rise to a limited library of shapes. For diblock copolymers, the most readily accessible morphologies are spheres on a cubic lattice, hexagonally packed cylinders or alternating lamellae. Especially for functional materials, a broader range of structures is desirable. Despite the control afforded by polymer synthesis^5^,^6^,^7^,^8^, it remains extremely challenging to use self-assembly to form arbitrary nanoscale shapes.

Here, we present methods for creating nanoscales meshes of arbitrary lattice symmetry. The approach relies critically on laser zone annealing (LZA), a photothermal technique we have recently developed^9^ to rapidly order self-assembling thin films. A focused laser line is swept across a film; the combination of high transient temperatures and large thermal gradients accelerates the kinetics of BCP self-assembly by >10^3^. Adding an elastic polymer cladding to the film induces simultaneous photoshear forces, owing to differential thermal expansion. These ‘soft-shear' (SS) effects contribute to the formation of uniaxially aligned BCP morphologies over macroscopic areas, after mere seconds of processing. This exceptional speed and control makes multistep fabrication practical. This work exploits this phenomenon to construct multi-layered nanomeshes. Aligned BCP morphologies are converted into rigid inorganic replicas, allowing additional BCP films to be applied and aligned independently. Thus, repeated SS-LZA allows lattice symmetry to be prescribed, based on the relative laser sweep directions between sequentially aligned BCP layers. We demonstrate that SS-LZA can generate nanoscale heterostructures of any lattice symmetry, and combining metals, insulators and semiconductors. With an eye towards functional nanomaterials, we provide electrical and optical characterization of these nanomeshes. This work lays the foundation for controlled fabrication of nanostructured thin films, achieving detailed control of the unit cell without sacrificing the speed and simplicity of self-assembly.

## Results

### Nanowire arrays

**Figure 1:**
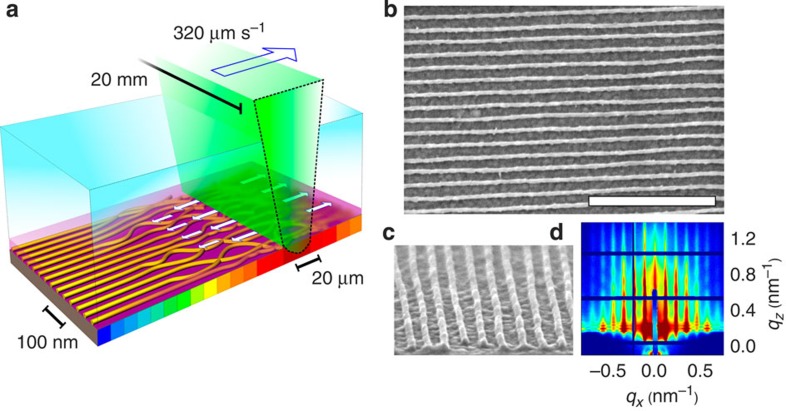
Soft-shear laser zone annealing (SS-LZA). SS-LZA is used to produce monolithically aligned block copolymer (BCP) templates for nanowire synthesis. (**a**) Experimental schematic: a focused laser line is absorbed by a layer of germanium underlying a BCP film, inducing local heating. The entire sample is rapidly annealed by sweeping the laser line. The temperature rise and steep thermal gradients induce accelerated self-assembly of the BCP cylindrical phase. Simultaneously, differential thermal expansion of a transparent PDMS cladding shears the BCP (white arrows), aligning the cylinders along the sweep direction. (**b**) Globally aligned array of nanowires after polymer to inorganic material conversion (scale bar 500 nm). (**c**) Cross-sectional view of the nanowires. (**d**) GISAXS of the polymer matrix after infusion with inorganic precursors (1 h soak, 0.02 M Na_2_PtCl_4_ in 0.5 M HCl).

In this work, we demonstrate how soft-shear laser zone annealing (Fig. 1a) can be applied repeatedly to order layers of BCP materials. Each layer is aligned using SS-LZA, and subsequently converted into an inorganic replica (Supplementary Fig. 1). A first ordered layer was constructed using SS-LZA to assemble thin films of a cylinder-forming polystyrene-*block*-poly(2-vinyl pyridine) (PS-*b*-P2VP) material. PS-*b*-P2VP provides a convenient and versatile platform for subsequent pattern conversion, due to the ease of selective infiltration of the P2VP block. The relatively high molecular weight (116 kg mol^–1^) polymer orders unacceptably slowly using traditional oven annealing, but requires only 27 s using photothermal processing. The aligned PS-*b*-P2VP cylindrical patterns were converted into metal nanowires using metal salt complexation of the P2VP block^10^,^11^. Preferential uptake of [PtCl_4_]^2–^ ions by P2VP strongly increases the X-ray scattering contrast, leading to the emergence of higher-order scattering peaks (>10 orders!), highlighting the excellent long-range positional order in the aligned polymer template (Fig. 1d). The polymer matrix was gently removed by oxygen plasma etching, and the resulting semiporous platinum nanowires (Fig. 1b,c) were thermally sintered to yield rigid, compacted nanowires (∼12 nm diameter). These nanowire arrays can be considered one-dimensional lattices (*p*2*mm* crystallographic symmetry).

### Nanomeshes

**Figure 2:**
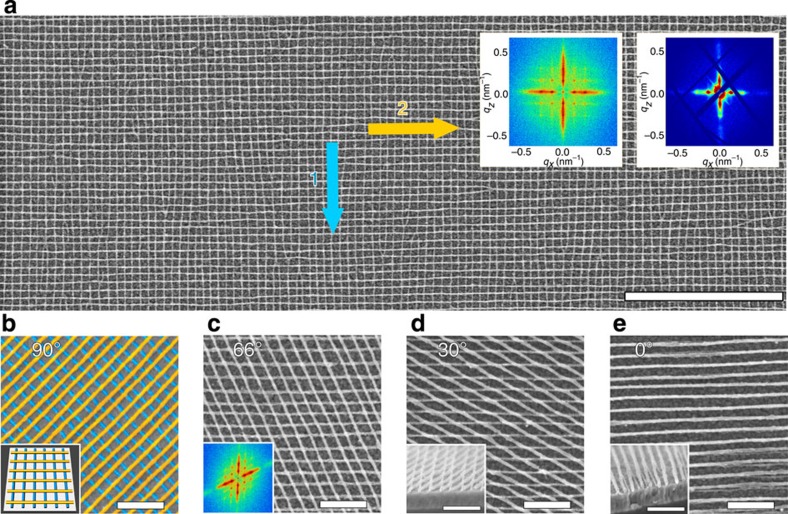
Nanomeshes made from two layers of block copolymer. SS-LZA allows fabrication of nanomaterials with morphologies not possible for conventional self-assembled diblock copolymers. In this example, double-layered arrays of nanowires with controlled hatching angle yield arbitrary rhombic lattices. (**a**) Top–down SEM image of a platinum grid prepared by two orthogonal SS-LZA steps (scale bar 1 μm). The insets show an FFT of the SEM (left) and the transmission X-ray diffraction pattern (right). (**b**–**e**) SEM images of various double-layered Pt nanomeshes (scale bars 200 nm). (**b**) False-coloured square lattice, with the first layer in blue and second in yellow. (**c**) Rhombic lattice obtained using a 66° angle between SS-LZA sweeps; inset shows an FFT of a lower magnification image. (**d**) 30° rhombic lattice with a cross-sectional SEM inset. (**e**) Two layers of the nanowires oriented in parallel direction tend to overlay; the cross-sectional SEM (inset) reveals the superimposed stack structure. (See Supplementary Figs 2–5 for wide area images.)

Repeating this photothermal BCP assembly and alignment on top of a nanowire array creates a layered nanostructure. Amazingly, photothermal shear fields are so strong that they can direct the orientation of the second BCP layer without interference from the underlying topography^12^,^13^. For example, we fabricate highly ordered square metallic grids (D_4_ point group; *p*4*mm* plane crystallographic group), spanning macroscopic area (>1 cm^2^), using a sequence of two orthogonal SS-LZA steps (Fig. 2a,b). The exceptional quality of the grids is evident from the sharp peaks in the Fourier transform (FFT) of the full scanning electron microscopy (SEM) images (Supplementary Figs 3–5). Transmission small-angle X-ray scattering measurements (spot size >10^4^ μm^2^) confirms that this order persists over macroscopic areas. The versatility of the presented method is further demonstrated by constructing rhombic metallic nanomeshes, where one can select any angle for the two photoalignment steps (Fig. 2c,d). This demonstrates the rhombic (centered-rectangular) Bravais lattice, with D_2_ point group symmetry (*c*2*mm* crystallographic group). Interestingly, an epitaxial effect is observed when the second BCP layer is aligned in the same direction as the first one, with the second layer overlapping on top of the first (Fig. 2e), likely due to preferential wetting interactions between P2VP and the Pt of the first layer.

**Figure 3:**
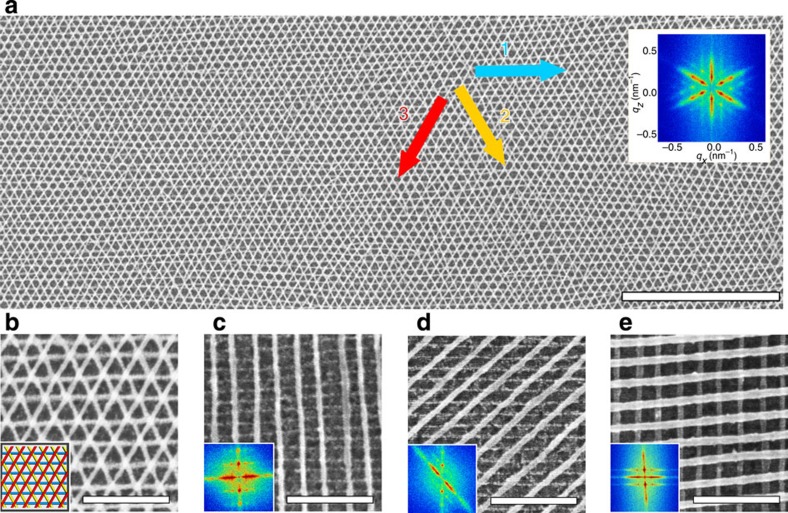
Complex nanostructures fabricated using SS-LZA. (**a**) Large-area SEM of a three-layered hexagonal lattice with all-60° crossing angles (scale bar 1 μm), with FFT inset. (**b**) SEM of a Pt triangular mesh. (**c**) Rectangular Pt lattice obtained using two BCPs with distinct molecular weights (*L*_0,first layer_=30 nm and *L*_0,second layer_=51 nm); FFT of the low magnification SEM inset. (**d**) Oblique lattice (45°), constructed with the same BCPs as in **c**. (**e**) Mixed composition nanomesh with metallic (Pt) bottom layer and metal oxide (Al_2_O_3_) top layer. (Scale bars in **b**–**e** are 200 nm.)

The hexagonal Bravais lattice was generated using three successive rounds of SS-LZA alignment and metallization (Fig. 3a). Equilateral triangles form when the nanowires in the third layer overlap with the intersections of the first two layers (Fig. 3b), exhibiting D_6_ point group symmetry (*p*6*mm* crystallographic group). Other registries of the third layer, with respect to the first two, represent different crystallographic symmetries within the hexagonal Bravais family.

The remaining possible two-dimensional (2D) Bravais lattices were constructed by combining BCPs of two distinct molecular weights. Specifically, grids of rectangles (Fig. 3c) and parallelograms (Fig. 3d) were obtained using a low-molecular weight PS-*b*-P2VP (*L*_0_=31 nm), followed by deposition of the previously described (*L*_0_*=*51 nm) material; the reverse order of layers yields similar results (not shown). These meshes demonstrate the rectangular Bravais lattice (D_2_ point group; *p*2*mm* crystallographic group) and the oblique Bravais lattice (C_2_ point group; *p*2 crystallographic group). Since the unit cell's angle and two lattice constants can be independently tuned, one can construct arbitrary 2D symmetries. In fact, as we have shown, we can construct all possible 2D Bravais lattices: square, oblique, rectangular, rhombic and hexagonal. These results can be contrasted to directed self-assembly of BCPs^14^,^15^, where lithographically defined topographic features^12^,^13^, chemical patterns^16^ or nanoscale confinement^17^ are used to locally control assembly^18^,^19^. Here, we use macroscopic fields to dictate the ultimate nanoscale unit cell, without compromising the inherent advantages of self-assembly: simple, rapid nanostructure formation over macroscopic areas. Our method can be combined with lithographic templating, with the shear direction selecting among degenerate ordering conditions. The diversity of structures can be further expanded by using BCP materials with alternate morphologies, such as rod–coil^6^, tri-block^7^ and multiblock^4^,^8^ copolymers, and blends of BCPs^20^,^21^,^22^,^23^,^24^. The versatility of SS-LZA enables any novel self-assembled morphology to be included in multicomponent nanostructures. Our method thus greatly broadens the diversity of BCP-derived structures^25^,^26^,^27^,^28^, generating them in a rapid and versatile manner.

### Heterostructures

A key advantage of the presented fabrication method is the independent control of structural symmetry and material composition. Since successive BCP layers can target different inorganic replicas, our method can naturally construct multicomponent nanostructures. For example, we created metal–insulator heterostructures composed of crossed Pt and Al_2_O_3_ nanowires (Fig. 3e). The metal oxide was formed by selectively infiltrating the second layer P2VP block with alumina, through vapour phase exposure to a metalorganic precursor, followed by water vapour^29^. It is possible to controllably grow the oxide structures, using sequential infiltration, conveniently tuning the nanomesh void dimensions. We have similarly prepared semiconducting ZnO nanostructures using vapour infiltration, and other metallic structures (Pd, Au and Rh) using liquid phase complexation of appropriate metal salts (Supplementary Fig. 6). Overall, the ability to control the composition of each layer of the nanostructure makes it possible to create hybrid structures that contain tailored mixtures of metals, insulators, and semiconductors.

### Nanomesh properties

**Figure 4:**
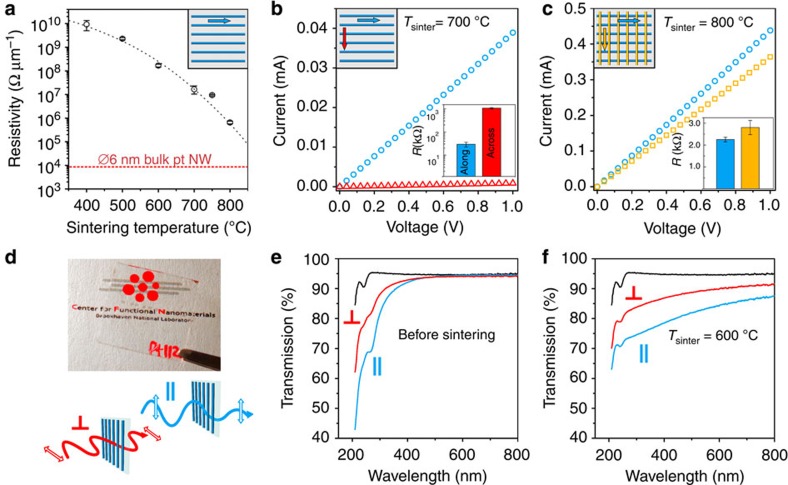
Electrical and optical properties of aligned Pt nanowires and square lattices. (**a**) Electrical resistivity (resistance per unit length of the wire) measured at room temperature as a function of sintering temperature (5 min in 5% H_2_ in Ar). Near 800 °C, resistivity approaches bulk Pt (dashed red line). Black-dashed line is a guide to the eye. (**b**) *I*–*V* characteristics of the single-layered nanowire arrays measured along the length of the nanowires (blue circles) and in the orthogonal direction (red triangles) display large resistance anisotropy. (**c**) *I*–*V* characteristics of the square grid; electrical properties are nearly isotropic. Error bars represent s.d. for *n*≥8 devices. (**d**) Photograph of a quartz substrate coated with aligned platinum nanowires, showing good transparency. A schematic of the two configurations of the polarizer-nanowires-analyzer used in optical anisotropy measurements is shown below. (**e**) Light transmission under normal incidence, with the nanowires long axis oriented with the incident electric field (blue curve) and against (red curve). Before sintering, the sample exhibits strong dichroism in the near-ultraviolet range. At longer wavelengths, the sample is highly transparent, similar to that of the uncoated substrate (fused quartz, black curve). (**f**) After thermal sintering, optical anisotropy extends across the visible spectrum.

This layer-by-layer method is ideally suited to fabricating nanomaterials with tunable functional properties. This control points towards applications as transparent electrodes, anti-reflection coatings and light management layers for improved solar harvesting or light emission. In order to elucidate the causal relationship between nanoscale symmetry and functional properties, we probed the electrical and optical response of the metallic nanowire arrays and nanomeshes. While the electrical conductivity of a single layer of Pt nanowires is initially poor, thermal sintering decreases their resistivity by ∼5 orders of magnitude (Fig. 4a), bringing them close to the response expected for bulk Pt (Supplementary Note 1). Measured along the nanowire axis, resistance scales linearly with wire length (Supplementary Fig. 7), confirming electrical continuity over distances exceeding 1,000 *L*_0_ (>50 μm). However, the nanowire arrays are strongly electrically anisotropic, exhibiting a >50 × higher resistance when measured across the nanowire axis. In contrast, the two-layer Pt nanomeshes are nearly electrically isotropic (Fig. 4c).The structural anisotropy of Pt wire arrays also manifests in anisotropic optical properties (Fig. 4d–f). On transparent substrates, nanowire arrays are dichroic in the ultraviolet, due to stronger light absorption when the incident electric field is aligned with the nanowire long axis. After sintering, the dichroism extends through the visible wavelength range (Fig. 4f). Corresponding reflectance-mode spectra of Pt nanowires also display optical anisotropy (Supplementary Fig. 8). Combined, these electrical and optical results highlight the causative role of the nanoscale lattice in determining functional properties.

## Discussion

In conclusion, we have demonstrated wide-area fabrication of multicomponent nanostructured thin films, with prescriptive control of nanoscale lattice symmetry and spacing. Laser zone annealing enables exceptionally rapid ordering of BCP films, with concomitant control of the alignment of the morphology. By combining rapid global patterning with precise local morphology control, SS-LZA makes multistep processing of self-assembling materials practical. We have exploited this technique, using multiple photoprocessing steps, combined with material conversion, to generate lattice symmetries that do not natively appear in the diblock copolymer phase diagram. The ability to tune the unit cell's angle and lattice constants independently allows for nearly arbitrary 2D symmetries. The formation of lines, squares, rhombuses, rectangles, parallelograms and triangles have been demonstrated. All 2D Bravais symmetries can thus be accessed. Independent control of the material of each layer enables construction of hybrid and functional nanostructures. This technique leverages the inherent power of self-assembly to create a well-defined periodicity spontaneously, while exploiting SS-LZA's ability to rapidly align this morphology over macroscopic areas. This paves the way towards yet more complex self-assembled nanoscale architectures.

## Methods

### Polymer films preparation

Substrates were 1-mm thick glass (BK-7 FisherFinest) or fused quartz (Ted Pella, GE-124) slides coated with 100 nm of germanium (Ar-plasma sputtered), which acts as a light-absorbing layer. Substrates were coated with 10 nm silicon nitride as a barrier layer (plasma-enhanced chemical vapor deposition (PECVD) grown at 350 °C). PS-*b*-P2VP of total molecular weight 116 kg mol^–1^ (79.0–36.5 kg mol^–1^, polydispersity index (PDI)=1.05) and volume fraction *f*_P2VP_=0.31 was obtained from Polymer Source, Inc. This material exhibits a cylinder-to-cylinder repeat spacing of *L*_0_=51 nm. The cylinder diameter is estimated to be *d*=30 nm, using *f*_P2VP_=(*π/*2✓3)(*d/L*_0_)^2^. Results are also shown for a 45 kg mol^–1^ PS-*b*-P2VP (32.5–12.0 kg mol^–1^, PDI=1.05), with *L*_0_=30 nm. Monolayer thick films of cylinder-forming BCP were prepared by spin casting from toluene solutions (1.5 and 1%, 2,000 r.p.m.). Films were dried under vacuum at 60 °C for 4 h to remove residual solvent^30^. After drying the samples were diced into 12 × 12 mm^2^ squares.

### Soft-shear laser zone annealing

A detailed description of the setup, including thermal field characterization, has been previously reported^9^. Briefly, a high power (3 W) solid-state green (532 nm) laser (Melles Griot 85 GHS 309) was used to irradiate the substrates. The beam was shaped by a set of lenses into a narrow line (20 μm full-width at half-maximum (FWHM) × ∼20 mm breadth) focused at the film surface. The laser line generates a thermal zone (90 μm FWHM), with a characteristic temperature *T*_HM_≈270 °C (where we characterize the annealing history using the temperature at the half-maximum of the thermal spike). We similarly define the total annealing time as the time above *T*_HM_. The narrow thermal zone creates sharp thermal gradients, ∇*T*_HM_≈1,500 °C mm^–1^ (the maximum thermal gradients near the peak of the thermal spike exceed 4,000 °C mm^–1^). The thermal gradients are responsible^31^,^32^ for greatly enhancing ordering kinetics. A thermostated baseplate was placed inside a vacuum chamber fitted with a transparent quartz window. The entire assembly sits on a motorized motion stage (Newport ILS 250CC), to allow sweeping the sample through the laser line.

For SS^33^ experiments, poly(dimethyl siloxane) pads (PDMS, Sylgard 184 with 5:1 mix ratio, vacuum cured 80 °C for 24 h) of 0.5 mm thickness were cut to match the size of the substrates and placed on top of the polymer films. The PDMS naturally achieves conformal contact with the polymer film. The samples were transferred into the vacuum LZA chamber and repeatedly swept through the laser line. A range of annealing protocols were investigated (Supplementary Fig. 9 and Supplementary Note 2). A typical protocol for the annealing of the monolayers consists of 64 sweeps at 320 μm s^–1^ (27 s total annealing). A shear field originates due to the large difference in the coefficient of thermal expansion between the polymer film (∼100 μm m^–1^ K^–1^) and the rigid substrate (∼1 μm m^–1^ K^–1^). This effect is further enhanced by the PDMS cladding (340 μm m^–1^ K^–1^), resulting in a large shear stress in the film acting in the ∇*T*-direction. By moving this shear zone through the sample, the BCP cylindrical domains become aligned in the sweep direction. The temperature of the sample holder baseplate was increased to 100 and 120 °C for glass and quartz substrates, respectively, to optimize the quality of the BCP alignment. The difference between the quartz and glass substrate processing conditions is an attempt to match thermal profiles experienced by the films on both materials, and a consequence of slightly higher heat conductivity of quartz. After annealing, the PDMS pads were gently peeled off; no evidence of residual PDMS was observed on the BCP films.

### Metallization

A P2VP block platinization protocol was adopted from Buriak and colleagues^10^,^11^, which involves immersion in a solution containing a metal salt. The pyridine residues of the P2VP block become protonated under acidic conditions, with subsequent complexation of negatively charged metal complex ions. In our implementation of this technique, SS-LZA aligned BCP films were immersed in a filtered solution of 20 mM Na_2_PtCl_4_ in 0.5 M HCl for 60 min (silicon nitride prevents the galvanic corrosion of underlying germanium), briefly washed in deionized water, and dried in an N_2_ stream. The duration of the soak in the metal precursor can, in principle, be used to control the diameter of the nanowires; however very short immersion inevitably yields defective nanowires with poor structural continuity. We allowed 60 min immersion to maximize complexation. The organic matrix was ashed in oxygen plasma (CS-1701, March RIE System, 100 mT, 20 W, 180 s) yielding porous inorganic nanowires. Subsequently, the nanowires were sintered using a rapid thermal processing (RTP) infrared oven (RTP-600S, Modular Process Technology Corp.) for 5 min at temperatures ranging from 400 to 800 °C, under reducing atmosphere (5% H_2_ in Ar). For samples processed at temperatures higher than 400 °C, fused quartz substrates were used rather than glass. A similar protocol with different metal ions ([PdCl]^2–^, [AuCl_4_]^–^ and [RhCl_6_]^3–^) was used to generate Pd, Au and Rh nanowires (Supplementary Fig. 6).

### Block-selective vapour infiltration

PS-*b*-P2VP templates were converted to metal oxide nanostructures by sequential infiltration synthesis^29^, in a commercial atomic layer deposition system (Cambridge Nanotech Savannah S100). Samples were exposed to four cycles of trimethylaluminum (TMA) (300 s, >5 torr) and water vapour (300 s, >5 torr) sequentially at 85 °C. TMA preferentially infiltrates P2VP domains, forming a Lewis acid–base complex; the water cycle initiates conversion to aluminium oxide. After infiltration, the remaining organic material was removed by oxygen plasma (20 W RF power, 100 mtorr) for 2 min. To convert the template into zinc oxide nanostructures, we replace TMA with diethylzinc and use similar sequential infiltration technique. In the case of zinc oxide, after the infiltration cycles, the remaining organic material was removed by annealing the sample in oxygen environment at 500 °C for 10 min.

### Nanomeshes

After the single-layered arrays of nanowires were sintered in the RTP oven at 400 °C to improve surface adhesion of the nanowires, the substrates were coated with a new layer of BCP using the same spin coating protocol. The SS-LZA and metallization procedures are also unmodified from those used in the first layer.

### Electrical measurements

After sintering of the nanostructures, several electrodes (Ti/Au 10 nm/100 nm thick) were patterned over the nanowires or nanomeshes using a conventional lithographic lift-off process. The electrodes were 3 μm × 30 μm parallel stripes spaced by 2 to 32 μm, with electrical leads connecting to macroscopic contact pads (Supplementary Figs 10 and 11, and Supplementary Note 3). Each device consisted of four electrode pairs oriented along the nanowire alignment direction and another four oriented in the perpendicular direction. The electrodes were briefly annealed after lift-off (400 °C for 5 min in RTP under H_2_/Ar atmosphere). For transparent conductors, the germanium layer was removed using a plasma etching procedure described below. Presented data were averaged over at least four independent devices. A miniature probe station (Signatone CM-170) and source-measure unit (Agilent 4156C) were used to acquire *I*–*V* characteristics for each electrode pair in 0–1 V biasing voltage range. Electrical resistivity per length of a single metallic nanowire was computed using in-parallel law assuming 51 nm spacing between the nanowires (calculated using FFT of SEM images) and the nominal distance between the probing electrodes.

### Optical measurements

Nanowire arrays were optically characterized both in reflection and in transmission mode using a variable angle spectroscopic ellipsometer (Woolam EC-400). For transmission measurements, the highly absorbing Ge layer (and SiN_*x*_ protecting layer) was removed by exposure to CHF_3_/O_2_ plasma (CS-1701, March RIE System, 25 sccm CHF_3_ and 5 sccm O_2_, 100 mT, 50 W, 90 s).

### Morphology characterization

The morphology of samples was characterized using SEM (Hitachi S-4800). High-resolution (2,560 × 1,920 pixels) top-view and cross-sectional images at a magnification of × 50,000 and × 100,000 were used for nanowire size measurements. FFT images were obtained from large area × 25,000 or × 10,000 images.

### Grazing incidence small-angle X-ray scattering

Grazing incidence small-angle X-ray scattering (GISAXS) measurements were performed at the X9 undulator beamline of the National Synchrotron Light Source at Brookhaven National Laboratory. Samples were measured under vacuum using an X-ray beam of 13.5 keV (*λ*=0.0918, nm). GISAXS data were collected across a range of incidence angles (0.07 to 0.20°). Silver behenate powder was used as a standard for data conversion to *q*-space.

## Additional information

**How to cite this article:** Majewski, P. W. *et al.* Arbitrary lattice symmetries via block copolymer nanomeshes. *Nat. Commun.* 6:7448 doi: 10.1038/ncomms8448 (2015).

## Supplementary Material

Supplementary Information Supplementary Figures 1-11, Supplementary Notes 1-3 and Supplementary References
