# Supplementary material for: Arbitrary lattice symmetries via block copolymer nanomeshes
Source: Nat Commun. 2015 Jun 23;6:7448. doi: 10.1038/ncomms8448 (PMC4557284; doi:10.1038/ncomms8448)
Supplement: Supplementary Information — Supplementary Figures 1-11, Supplementary Notes 1-3 and Supplementary References [file ncomms8448-s1.pdf]

Supplementary Figure 1

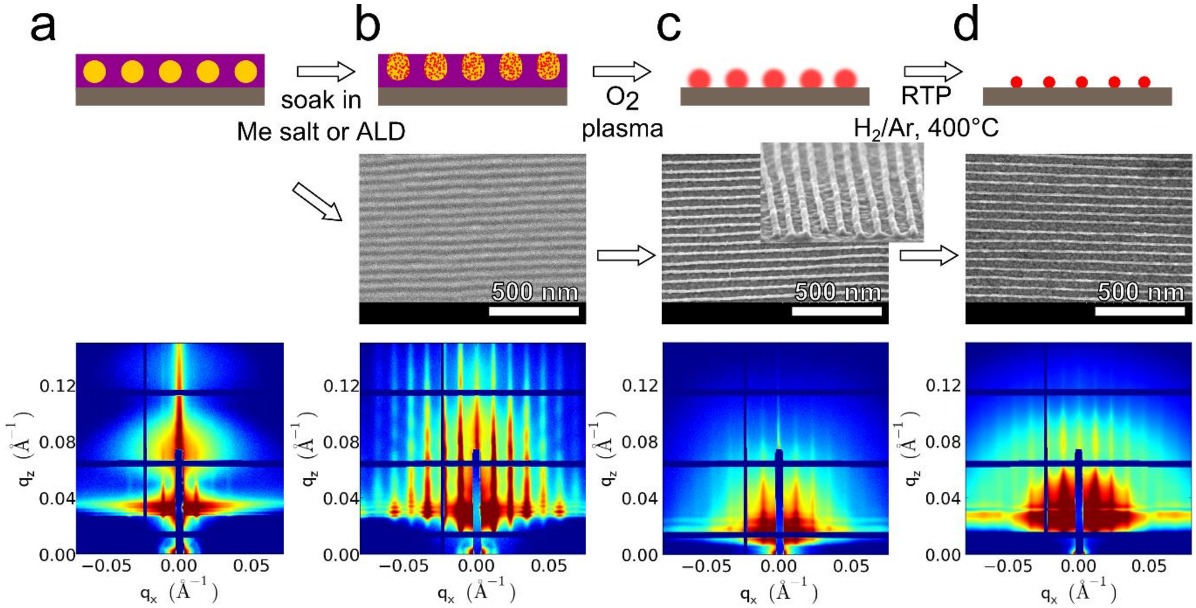

Supplementary Figure 1. Templated synthesis of globally-aligned nanowires arrays. Top-row sequence is a schematic of the polymer-to-inorganic nanomaterial conversion. Middle and bottom rows contain SEM images and GISAXS diffractograms acquired at each synthetic stage. **a**, SS-LZA aligned PS-*b*-P2VP thin film in the native state (SEM not shown due to poor imaging contrast). **b,** Infusion of the P2VP block with inorganic precursors delivered in solution (1 hr soak, 0.02 M Na_2PtCl_4 in 0.5 M HCl) or in gaseous form (ALD process). **c,** Polymer matrix removal by oxygen plasma. **d,** Sintering of nanowires by rapid thermal processing under reducing atmosphere.

Supplementary Figure 2

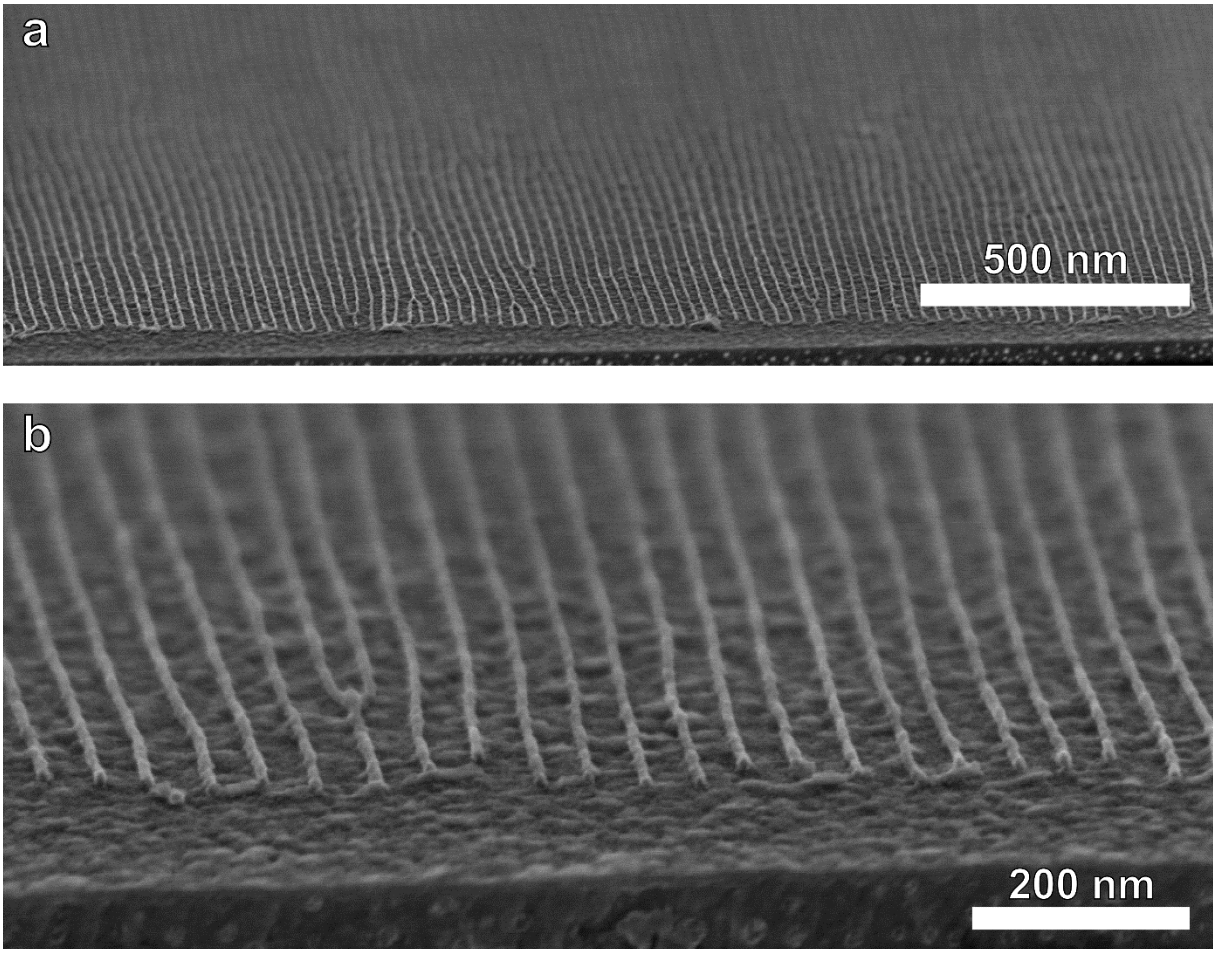

Supplementary Figure 2. Cross-sectional SEM images of single-layered array at 25,000× (**a**) and 100,000× (**b**).

Supplementary Figure 3

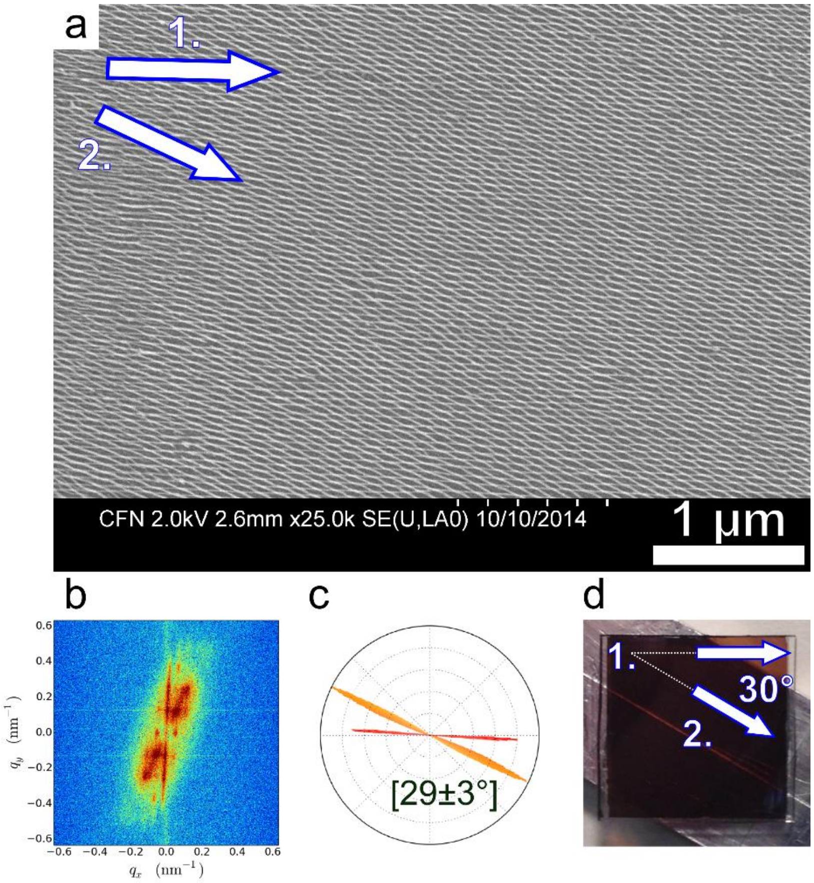

Supplementary Figure 3. Pt nano-mesh with 30°-crossing angle. a, Large area SEM image of the mesh. **b,** Fast Fourier transform (FFT) of the SEM. **c,** Azimuthal histogram of the nanowire orientation obtained by radial integration of the FFT in the annular region surrounding the 100 correlation signal. The average crossing-angle value and its standard deviation were calculated from multiple spots on the sample. **d,** A photograph illustrating the macroscopic orientation of the substrate on the holder. The nano-mesh was obtained by two subsequent metallizations of the aligned S2VP 116 kg mol^{-1} template.

Supplementary Figure 4

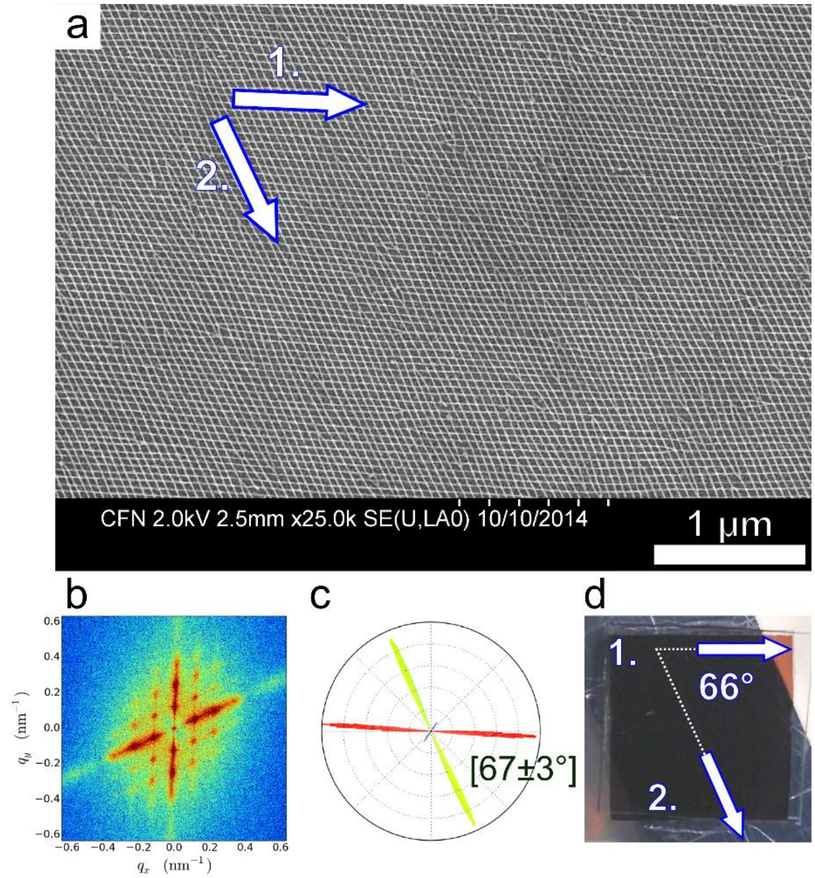

Supplementary Figure 4. Pt nano-mesh with 66°-crossing angle. a, Large area SEM image of the mesh. **b,** Fast Fourier transform (FFT) of the SEM. **c,** Azimuthal histogram of the nanowire orientation obtained by radial integration of the FFT in the annular region surrounding the 100 correlation signal. The average crossing-angle value and its standard deviation were calculated from multiple spots on the sample. **d,** A photograph illustrating the macroscopic orientation of the substrate on the holder. The nano-mesh was obtained by two subsequent metallizations of the aligned S2VP 116 kg mol^{-1} template.

Supplementary Figure 5

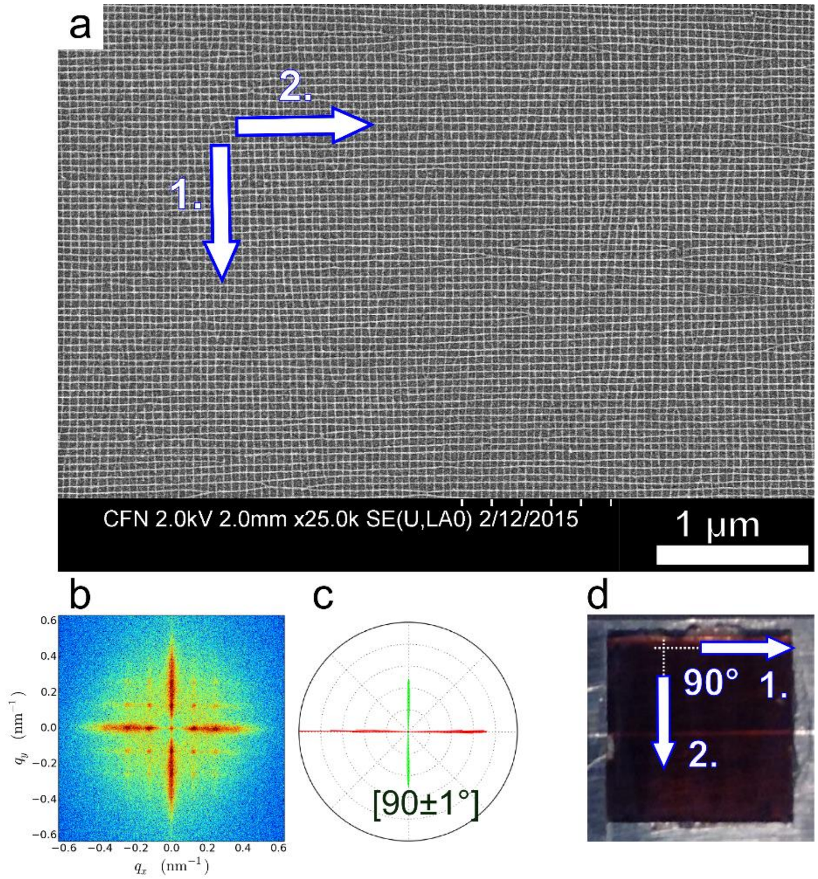

Supplementary Figure 5. Pt nano-mesh with 90°-crossing angle. a, Large area SEM image of the mesh. **b,** Fast Fourier transform (FFT) of the SEM. **c,** Azimuthal histogram of the nanowire orientation obtained by radial integration of the FFT in the annular region surrounding the 100 correlation signal. The average crossing-angle value and its standard deviation were calculated from multiple spots on the sample. **d,** A photograph illustrating the macroscopic orientation of the substrate on the holder. The nano-mesh was obtained by two subsequent metallizations of the aligned S2VP 116 kg mol^{-1} template.

Supplementary Figure 6

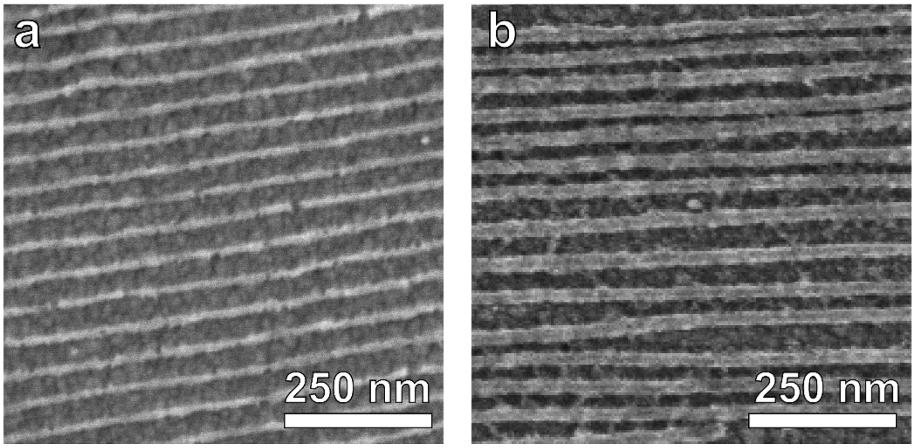

Supplementary Figure 6. Arrays of inorganic nanowires aligned by SS-LZA. a, Palladium nanowires synthesized by aqueous precursor-delivery method. **b,** ZnO nanowires synthesized by 4 ALD infusion cycles ($Zn(CH_3)_2$ precursor).

Supplementary Figure 7

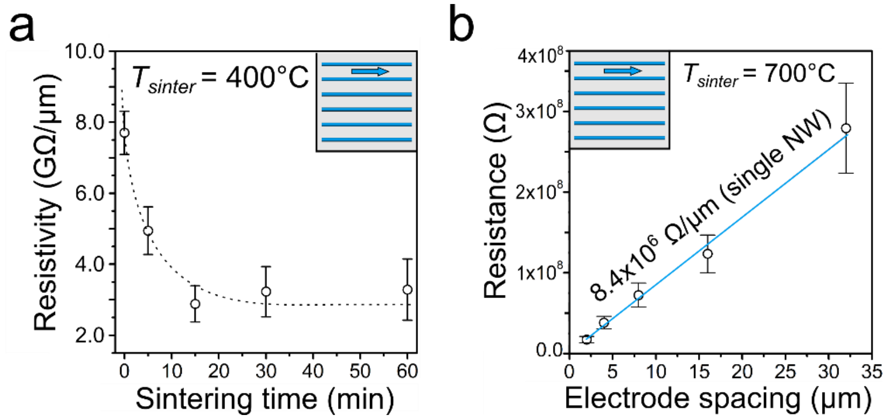

Supplementary Figure 7. Electrical characterization of the nanowire arrays. a, Electrical resistivity as a function of sintering time. Resistivity drops rapidly with time, even at low sintering temperatures (400 °C). **b,** Sintered nanowires exhibit Ohmic conductance, with resistance scaling linearly with electrode spacing.

Supplementary Figure 8

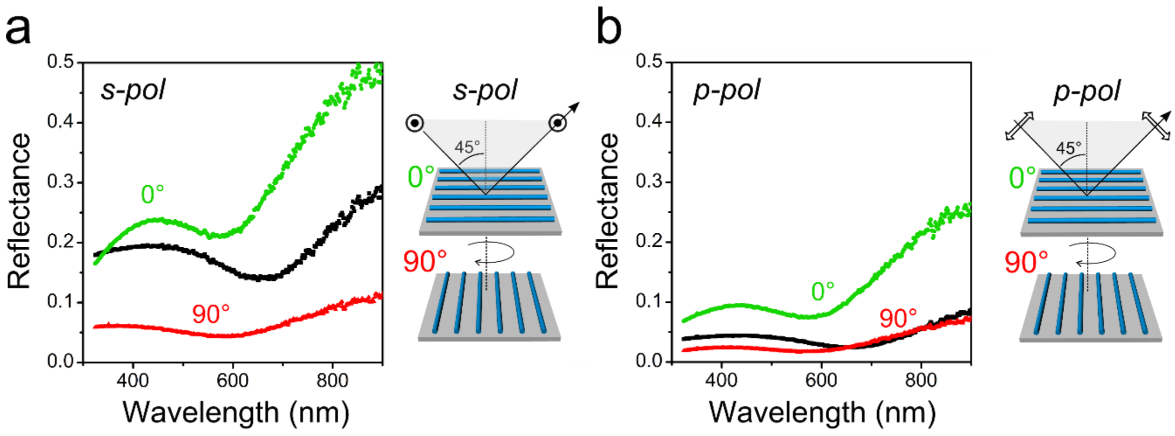

Supplementary Figure 8. Optical characterization of the nanowire arrays on reflective 100 nm Ge/ 10 nm Si_3N_4 substrates. Ellipsometric reflectance spectra collected at 45° angle of incidence. **a,** Reflectance of s-polarized light (polarized perpendicular to the plane-of-incidence). **b,** Reflectance of p-polarized light (polarized in the plane-of-incidence) The curves denoted as 0° and 90° correspond to parallel and perpendicular orientations of the nanowire array with respect to the plane-of-incidence. The spectra were collected after thermal sintering at 400 °C for 5 min in 5% H_2/Ar.

Supplementary Figure 9

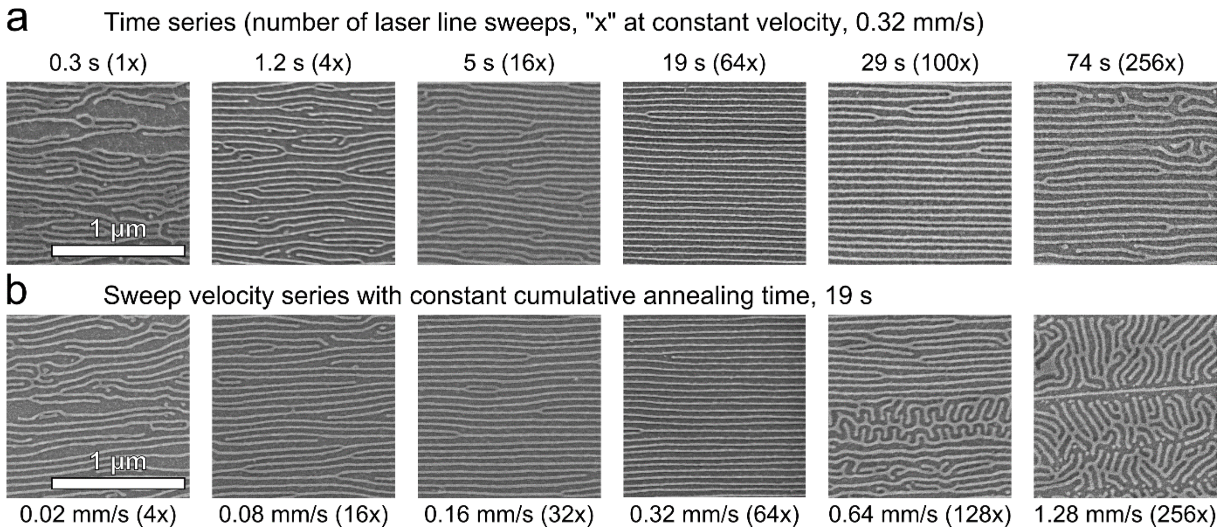

Supplementary Figure 9. Influence of the number of laser-line sweeps and the sweep velocity on the morphology of NW arrays. a, The number of sweeps controls the effective local annealing time of the BCP. After approximately 50 sweeps at 0.32 mm s^{-1}, morphology alignment saturates (with further sweeps in fact somewhat worsening order). **b,** Sweep velocity series, where cumulative annealing time is kept constant. Sweep velocity, affecting the shear-rate in SS-LZA, influences the morphology of the film. Low shear-rate yields poorly ordered arrays, while excessive speeds evidently induce non-equilibrium comb-like patterns. Presented images (1.2×1.2 μm^2) were cropped from SEM images collected at 50,000× magnification after metallization of the S2VP 116 kg mol^{-1} template. The sweep-direction is horizontal in all images.

Supplementary Figure 10

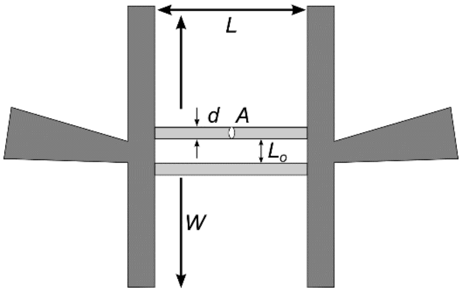

Supplementary Figure 10. Schematic of electrodes in rectangular-gap configuration aligned along NWs direction.

Supplementary Figure 11

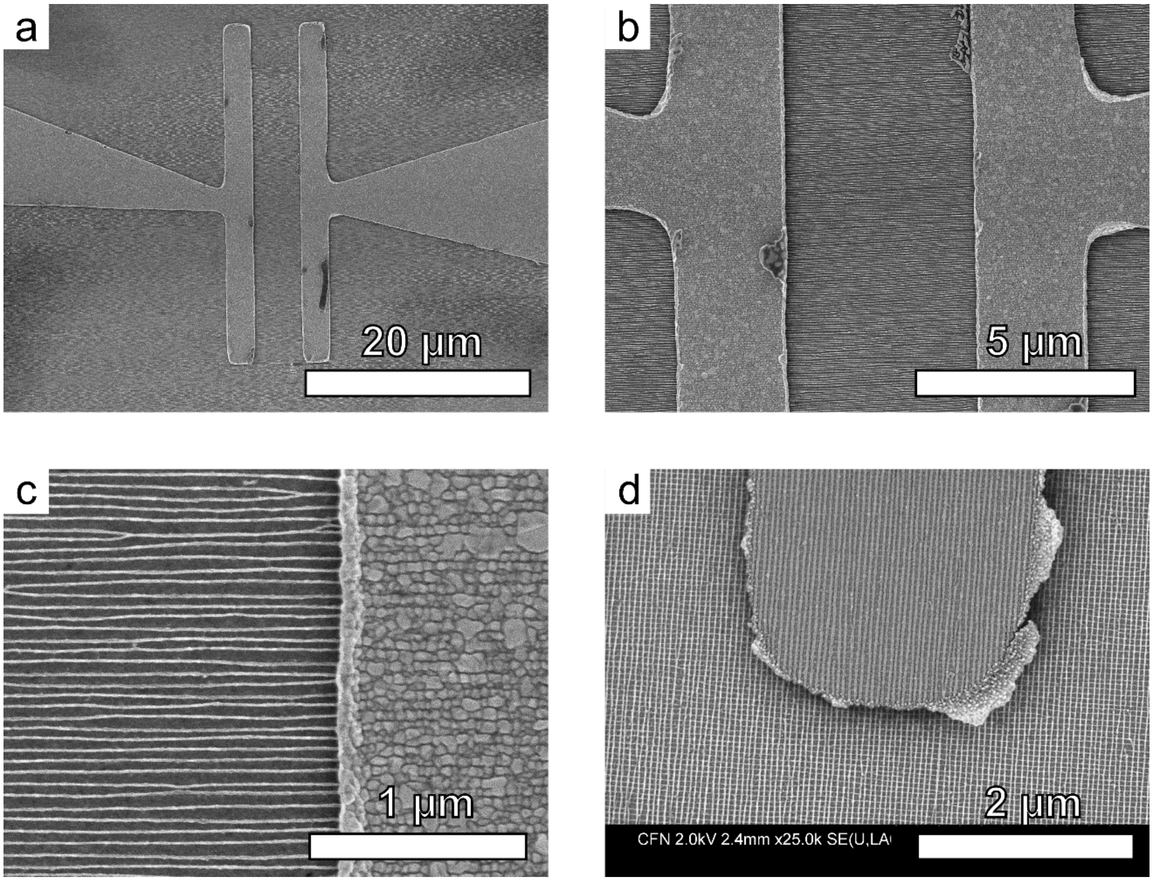

Supplementary Figure 11. Ti/Au electrodes deposited on the nanowire arrays by thermal evaporation through a photolithographic mask. SEM images of single-layered array at 2,500× (**a**), 10,000× (**b**), and 50,000× (**c**). **d,** 90°-nanomesh. If the thickness of the electrode is thinner than approx. 70 nm, the top surface of the electrode replicates the topography of the mesh. This demonstrates another viable mode of material conversion.

Supplementary Note 1: Theoretical limit for electrical resistance of Pt nanowires derived from PS-*b*-P2VP 116 kg mol^{-1} template

The diameter, d_{P2VP}, of the P2VP cylindrical domains can be calculated from the cylinder center-to-center distance, L_o = 51 nm, and the volume fraction of the P2VP block, φ_{P2VP} = 0.306, using the following relationship $\varphi_{P2VP} = (\pi/2\sqrt{3})(d/L_0)^2$. After algebraic rearrangement and numerical values substitutions:

$$d_{P2VP} = \left(\frac{2\sqrt{3}}{\pi}\varphi_{P2VP}\right)^{1/2} \cdot L_o \cong 30\, nm$$

The diameter of the platinum nanowire derived from the template, $d_{Pt\,NW}$, can be calculated from:

$$d_{Pt\,NW} = \left(C.R. \ \cdot \frac{\rho_{P2VP}}{\rho_{Pt}} \cdot \frac{M_{Pt}}{M_{2VP}}\right)^{1/2} \cdot d_{P2VP},$$

where we use the experimental values of[1, 2] ρ_{Pt},= 21.45 g cm^{-3}, M_{Pt} = 195.1 g mol^{-1}; and ρ_{P2VP} = 1.14 g cm^{-3}, M_{2VP} = 105.1 g mol^{-1} are densities and molecular weights of Pt and P2VP, respectively. *C.R.* is the molar complexation ratio of Pt to pyridyl groups. Under the assumption that the nanowires achieve the bulk metal density and a maximum complexation ratio *C.R.* = 1, $d_{Pt\,NW}$ = 9.4 nm. According to some reports[3], 2 or more 2VP ligands are complexing each Pt atom. Using *C.R.* = 0.4 (1:2.5) results in $d_{Pt\,NW}$ = 5.8 nm.

Electrical resistance, R_{NW}, of a single 5.8 nm-thick Pt nanowire with a length of *L* can be calculated using electrical resistivity of metallic Pt, $\rho_{ele\,Pt} = 1.07 \times 10^{-7}$ Ω·m[1]:

$$R_{NW} = \rho_{ele\ Pt} \frac{1}{\pi} \frac{L}{\left(\frac{d_{Pt}}{2}\right)^2} \cong 4.1 \times 10^3 \cdot L \left[\frac{\Omega}{\mu m}\right]$$

Electrical resistivity (resistance per 1 µm length), $R_{NW/\mu m}$ is equal to 4.1×10^3 Ω μm^{-1}. Square resistance of an array of the nanowires made of bulk Pt is:

$$R_{array} = R_{NW/\mu m} \frac{L}{W/L_o} = R_{NW/\mu m} \frac{1 \mu m}{1\ \mu m / 0.051 \mu m} \cong 207\, \Omega/\text{sq.}$$

for 1:1 *C.R.*

Supplementary Note 2

The degree polymer alignment *via* SS-LZA is sensitive to the shear-rate, with stronger alignment arising from laser-line sweep velocities >100 µm s^{-1}. Multiple sweeps through the thermal zone progressively improves order and alignment, as evidenced by the appearance of higher-order peaks in synchrotron GISAXS measurements, and a concomitant reduction of dislocation defects observed in SEM images (Supplementary Figure 9a). By comparison, lower molecular weight materials can typically be equivalently ordered with a single SS-LZA sweep. For the 116 kg mol^{-1} material, the alignment saturates after ~50 laser passages and, interestingly, deteriorates beyond this point, especially if high sweep velocities (≥ 640 µm s^{-1}) are employed (Supplementary Figure 9b). Our optimized protocol, used throughout this study, consisted of 64 sweeps at 320 µm s^{-1}. This corresponds to a total local annealing (time above T_{HM}) of only 27 s, emphasizing the extremely rapid assembly.

We note that the large molecular weights used herein (116 kg mol^{-1}) are generally considered extremely challenging to order; e.g. oven-annealing for hours or even days would yield small grain sizes. Furthermore, we observed that oven-annealing (24 hr at 200 °C) of this material on conventional hydrophilic substrates (silicon, glass, silicon nitride) gave rise to poorly-ordered perpendicular (vertical) cylinder orientations, rather than parallel (in-plane) structures. These materials thus require special approaches to achieve reasonable order. SS-LZA is uniquely suitable to process inherently problematic systems: it shortens processing times by several orders-of-magnitude, and provides means of controlling both the out-of-plane orientation (vertical *vs.* horizontal) and in-plane alignment of the morphology.

Supplementary Note 3: The scaling of electrical resistance of the aligned array with the molecular weight (MW) of the polymer used as a template

Consider a rectangular-gap electrode configuration (Supplementary Figure 10) where L is the distance between the electrodes, and W is their width. If the resistance of a single aligned nanowire spanning between the electrodes is R_{NW}, the resistance of the array R_{array} consisting of n nanowires can be expressed, using the in-parallel law of conduction, as:

$$R_{array} = \frac{R_{NW}}{n} \tag{1}$$

R_{NW} is proportional to the electrical resistivity of the metal, ρ, the nanowire length (L) and inversely proportional to the cross-sectional area of the nanowire, A.

$$R_{NW} = \rho \frac{L}{A} \tag{2}$$

The number of nanowires per width of electrode is equal to W/L_o, where L_o is the cylinder center-to-center distance in the BCP monolayer. Combining the above equations yields:

$$R_{array} = \rho \frac{L}{W} \frac{L_o}{A} \tag{3}$$

Assuming a power-law scaling of L_o ~ $MW^{2/3}$ (where MW is molecular weight of the BCP) and a quadratic relationship between A and L_o, R_{array} scales with MW as:

$$\boldsymbol{R_{array}} \propto \rho \frac{L}{W} \frac{MW^{2/3}}{\left(MW^{2/3}\right)^2} = \rho \frac{L}{W} \frac{MW^{2/3}}{MW^{4/3}} = \rho \frac{L}{W} \boldsymbol{MW^{-2/3}} \tag{4}$$

It is thus advantageous to use high MW templates if high electrical conductivity of the nanowire array is desired.

Supplementary References

1. in CRC Handbook of Chemistry and Physics, 84th Edition (ed. Lide, D.R.) (2003).
2. Zha, W., Han, C.D., Lee, D.H., Han, S.H., Kim, J.K., Kang, J.H. & Park, C. Origin of the Difference in Order−Disorder Transition Temperature between Polystyrene-block-poly(2-vinylpyridine) and Polystyrene-block-poly(4-vinylpyridine) Copolymers. *Macromol.* **40**, 2109-2119 (2007).
3. Saha, C.R. & Bhattacharya, S. Reduction of nitroaromatics with poly(vinylpyridine) complexes of palladium(II) and platinum(II). *J. Chem. Technol. Biotechnol.* **37**, 233-245 (1987).
